# A clinical study on the screening efficacy of six thrombotic markers TAT, PIC, TM, t-PAI-C, D-D, and FDP for hypercoagulable state in pregnant women

**DOI:** 10.12669/pjms.41.1.9755

**Published:** 2025-01

**Authors:** Tao Yu, Wenjuan Zhang, Yunshuang Wang, Ran Chen, Shuo Luo

**Affiliations:** 1 Tao Yu Clinical Laboratory, Baoding Maternal and Child Health Hospital, Baoding 071000, Hebei, China; 2 Wenjuan Zhang Clinical Laboratory, Baoding Maternal and Child Health Hospital, Baoding 071000, Hebei, China; 3 Yunshuang Wang Clinical Laboratory, Baoding Maternal and Child Health Hospital, Baoding 071000, Hebei, China; 4 Ran Chen Clinical Laboratory, Baoding Maternal and Child Health Hospital, Baoding 071000, Hebei, China; 5 Shuo Luo High-risk Obstetrics, Baoding Maternal and Child Health Hospital, Baoding 071000, Hebei, China

**Keywords:** Pregnant women, Hypercoagulable state, Thrombotic marker, Screening, Hemagglutination

## Abstract

**Objective:**

To investigate the screening efficacy of six thrombotic markers for hypercoagulable state (HCS) in pregnant women, including thrombin–antithrombin III complex (TAT), plasmin-alpha-2 plasmin inhibitor complex (PIC), thrombomodulin (TM), tissue-type plasminogen activator inhibitor complex(t-PAI-C), D-dimer(D-D), and fibrinogen degradation products (FDP).

**Methods:**

This was a retrospective study. Eighty-five high-risk pregnant women who underwent antenatal examination at Baoding maternal and Child Health Hospital from December 2022 to September 2023 were included as the observation group, while 85 healthy pregnant women without complications or comorbidities who underwent routine antenatal examinations at our hospital were randomly enrolled as the control group. Analyzed the screening value of these six thrombotic markers in pregnant women at risk of HCS.

**Results:**

The levels of TAT, PIC, TM, FDP, and D-D in the observation group were significantly higher than in the control group (*P* < 0.05, respectively). Pearson correlation analysis showed a weak correlation between maternal age and TAT levels (*r* = -0.179, *P*< 0.05) as well as between BMI and FDP and D-D (*r* = 0.214, 0.232, *P*< 0.05, respectively). Binary logistic regression analysis revealed that abnormal D-D levels were a risk factor for HCS in pregnant women (OR = 0.117, 95% CI: 0.621–0.639, *P*< 0.05). The area under the joint curve was 0.757, indicating that the model is of certain predictive value for HCS in pregnant women.

**Conclusion:**

TAT, PIC, TM, t-PAI-C, D-D, and FDP can used for the screening of HCS in pregnant women and have a certain predictive value for high-risk pregnant women.

## INTRODUCTION

Hypercoagulable state (HCS) in pregnant women is one of the common complications during pregnancy and delivery due to physiological and pathological factors during pregnancy and childbirth, which can lead to enhanced coagulation and limited fibrinolysis. HCS is prone to the formation of blood clots, which may increase the risk of thrombosis in pregnant women and adversely affect the health of both the mother and the fetus.^1^,^2^ To assess HCS in pregnant women, clinicians typically rely on markers of hemostasis and thrombosis. Among these markers, thrombin-antithrombin III complex (TAT) is a marker of thrombin generation, reflecting the degree of activation of the coagulation system.^3^

Plasmin-alpha-2 plasmin inhibitor complex (PIC) serves as a marker of platelet activation indicative of platelet activity and postoperative bleeding status.^4^ Thrombomodulin (TM) is a thrombomodulatory protein involved in the inhibition of the coagulation process.^5^ Tissue-type plasminogen activator inhibitor complex (t-PAI-C) is a complex of tissue-type plasminogen activator inhibitor reflecting the degree of fibrinolysis inhibition. In the clinical setting, t-PAI-C can be used for the preliminary assessment of high-risk conditions like preeclampsia in pregnant women.^6^ D-dimer (D-D) and fibrinogen degradation products (FDP) are products of thrombus degradation, and their elevation can indicate thrombus formation and decreased fibrinolytic activity.^7^,^8^ This study investigated the screening efficacy of six thrombotic markers for HCS in pregnant women, including TAT, plasmin-alpha-2 PIC, TM, t-PAI-C, D-D, and FDP, thereby assisting clinicians in evaluating the risk of hypercoagulable state and developing individualized treatment plans.

## METHODS

This was a retrospective study. Eighty five high risk pregnant women who underwent antenatal examination at Baoding maternal and Child Health Hospital from December 2022 to September 2023 were included as the observation group, while 85 healthy pregnant women without complications or comorbidities who underwent routine antenatal examinations at our hospital were randomly enrolled as the control group. Pregnant data including demographic data were retrieved from electronic medical record systems of our hospital. General information for both groups is shown in Table-I.

**Table-I:** Comparison of general information between the two groups.

Group	Age (years)	Gestational age (weeks)	BMI (kg/m^2^)	Hypertension	

				Yes	No
Observation group (*n* = 85)	28.13±5.80	38.46±1.13	32.57±7.43	13	72
Control group (*n* = 80)	27.68±6.10	38.76±1.08	30.11±5.45	0	80
*t/χ^2^*	0.490	1.772	2.408	13.282	
*p*	0.625	0.078	0.017	<0.001	

### Ethical Approval

The study was approved by the Institutional Ethics Committee of Baoding Maternal and Child Health Hospital (No.:2023-01-K007; date: May 12, 2023), and written informed consent was obtained from all participants and their families.

### Inclusion criteria for the observation group:

- Undergoing blood tests of the six thrombotic markers at Baoding maternal and Child Health Hospital;
- meeting the criteria for high-risk pregnant women as described in Obstetrics and Gynecology^9^: A) Advanced maternal age (age > 35 years) or aged 20–35 with a history of hypertension; B) Pregnant women at 20 weeks with systolic blood pressure > 140 mmHg and diastolic blood pressure > 90 mmHg, or those with continuously increasing blood pressure during pregnancy, with at least 1+ or 2+ proteinuria in two consecutive dipstick urinalyses; C) Blood pressure during pregnancy rising by more than 30 mmHg compared to baseline, with at least 1+ proteinuria; D) Obese pregnant women with a body mass index (BMI) > 40;
- No history of diabetes, hyperthyroidism, or other underlying diseases or complications, and no conditions leading to coagulation dysfunction.

### Inclusion criteria for the control group:

- Undergoing blood tests of the six thrombotic markers at Baoding maternal and Child Health Hospital.
- Fulfilling the following criteria: A) Pregnant women aged 20-35 years without high-risk factors or a history of hypertension; B) Pregnant women at 20 weeks with systolic blood pressure < 140 mmHg and diastolic blood pressure < 90 mmHg.
- Absence of other underlying diseases or complications, and conditions leading to coagulation dysfunction.
- Exclusion criteria for all research subjects
- Incomplete clinical data.
- Severe liver or kidney dysfunction or neurological diseases.
- Comorbid conditions affecting the hematological system or coagulation function (skin lesions, submucosal bleeding, gingival bleeding, gastrointestinal or other visceral bleeding).
- Other underlying diseases or complications.

### Methods:

When the participants were in a basal state, 3mL of ulnar vein blood was drawn into citrate tubes. After thorough mixing, the blood samples were centrifuged at 3500 rpm for 15 minutes, and the plasma was harvested as lipemia-free samples for future use. Thrombotic markers, including TAT, PIC, TM, t-PAI-C, D-D, and FDP, were detected using the Wondfu Shine i2900 full-automatic chemiluminescence detection platform and corresponding reagent kits, as well as quality controls. According to the standards outlined in the clinical laboratory handbook, the reference ranges for these markers are specified as follows: TAT (0–4.08 ng/mL), PIC (0–0.85 mg/L), TM (3.82–13.85 TU/ml), t-PAI-C (0–10.52 ng/mL), D-D (0–0.5 mg/L), and FDP (0–5 mg/L).

### Statistical analysis:

The software SPSS 22.0 was used for data processing. Enumeration data were presented as “frequency (percentage) [*n*(%)]” and comparisons were analyzed using the chi-square (*χ^2^*) test. Measurement data following normal distributions were expressed as “mean ± standard deviation (*χ̅*±*S*)” and examined by the t-test. Non-normally distributed measurement data were represented by “median (1^st^ quartile, 3^rd^ quartile) [*m*(*q_1_*, *q_3_*)]” and comparisons were assessed using non-parametric tests. Pearson correlation analysis was conducted to assess the correlations of baseline data (such as maternal age, gestational age, and BMI) with the thrombotic markers, where *r* ≥ 0.8 indicated a very strong correlation, 0.6 ≤ *r* < 0.8 suggested a strong correlation, 0.4 ≤ *r* < 0.6 showed a moderate correlation, 0.2 ≤ *r* < 0.4 implied a weak correlation, and *r* < 0.2 reflected a very weak or no correlation at all. Binary logistic regression analysis was performed to assess the impact of the thrombotic markers on high-risk pregnant women. Receiver Operating Characteristic (ROC) curve analysis was conducted to evaluate the predictive accuracy of the thrombotic markers for high-risk pregnant women. An area under the curve (AUC) of 0.5 to 0.7 (excluding) indicated relatively low accuracy, 0.7 to 0.9 (excluding) indicated moderate accuracy, and ≥ 0.9 indicated high accuracy. *P*< 0.05 was considered statistically significant.

## RESULTS

The two groups did not differ greatly in t-PAI-C levels (*P* > 0.05). In contrast, the levels of TAT, PIC, TM, FDP, and D-D in the observation group were significantly higher than in the control group (*P* < 0.05, respectively), Table-II. The results of Pearson correlation analysis revealed a weak correlation between maternal age and TAT levels (*P* < 0.05). Additionally, BMI was weakly correlated with FDP and D-D (*P* < 0.05, respectively), Table-III.

**Table-II:** Comparison of thrombotic markers between the two groups [m(q1, q3)].

Group	Case (n)	TAT	PIC	TM	t-PAIC	FDP	D-D
Observation group	85	3.37(1.91,9.52)	0.25(0.18,0.37)	5.85(4.15,7.67)	5.05(2.82,6.70)	2.30(2.30,2.72)	0.24(0.22,0.54)
Control group	80	1.45(0.79,3.83)	0.14(0.13,0.27)	5.20(2.33,6.69)	3.78(1.95,6.59)	2.30(2.30,2.31)	0.22(0.22,0.27)
*Z*		-4.079	-4.059	-2.339	-1.720	-2.790	-3.482
*p*		<0.001	<0.001	0.019	0.085	0.005	<0.001

**Table-III:** Correlations between basic information and thrombotic markers.

		TAT	PIC	TM	t-PAIC	FDP	D-D
Age	*r*	-0.179	-0.130	-0.135	0.012	0.131	0.150
	*p*	0.021	0.096	0.083	0.878	0.094	0.055
Gestational age (weeks)	*r*	-0.080	0.103	0.006	0.028	-0.025	-0.062
	*p*	0.306	0.187	0.937	0.725	0.753	0.430
BMI	*r*	-0.095	0.077	0.034	-0.005	0.214	0.232
	*p*	0.223	0.324	0.667	0.945	0.006	0.003

Based on the results of the univariate analysis, a binary logistic regression analysis was conducted, with group (*1* = observation group, *2* = control group) as the dependent variable and statistically significant parameters as independent variables. The results indicated that abnormal D-D levels were a risk factor for HCS in pregnant women (*P* < 0.05), Table-IV.

**Table-IV:** Influencing factors for HCS in high-risk pregnant women.

Variable	B	SE	Wald	p	OR	95% CI
TAT	-0.049	0.032	2.310	0.129	0.952	0.893–1.014
PIC	-0.904	0.651	1.928	0.165	0.405	0.113–1.451
TM	-0.102	0.073	1.954	0.162	0.903	0.782–1.042
FDP	0.030	0.170	0.032	0.859	1.031	0.739–1.438
D-D	-2.147	0.867	6.136	0.013	0.117	0.621–0.639
Constant	1.700	0.604	7.917	0.005	5.477	

According to the logistic regression results, the predictive model index was calculated as “-0.049*TAT - 0.904*PIC - 0.102*TM + 0.030*FDP - 2.147*D + 1.700”. By substituting variables into the equation, an ROC curve was plotted. The results showed that the AUC (joint curve) of this model was 0.757, indicating a certain predictive value for HCS in high-risk pregnant women, Table-V.

**Table-V:** Predictive model analysis.

Item	AUC	SE	p-value	95% CI
TAT	0.684	0.042	<0.101	0.601–0.767
PIC	0.683	0.042	<0.001	0.601–0.765
TM	0.605	0.044	0.019	0.519–0.692
FDP	0.608	0.044	0.016	0.523–0.694
D-D	0.653	0.042	0.001	0.570–0.736
AUC (joint curve)	0.757	0.038	0.000	0.683–0.831

## DISCUSSION

TAT, PIC, TM, FDP, D-D, and t-PAIC are common thrombotic markers in clinical practice. In this study, the levels of TAT, PIC, TM, FDP, and D-D in high-risk pregnant women were significantly higher than in the healthy control group, but the difference in t-PAIC levels between the two groups lacked statistical significance. Preliminary analysis suggests that hormonal changes and hemodynamic alterations in healthy pregnant women may lead to increased activity of the coagulation system to prevent hemorrhage during childbirth. Following activation of the coagulation system, changes also occur in the fibrinolytic system, resulting in alterations in the thrombotic markers. After delivery, the coagulation system undergoes adjustments and resumes normal. However, for high-risk pregnant women, preexisting conditions affecting coagulation or complications during pregnancy (such as gestational hypertension and preeclampsia) entail an increased risk of thrombosis in the mother. Abnormalities in thrombotic markers can manifest through the coagulation status, further affecting other physiological parameters and pathological changes.^10^,^11^ For instance, the study by Kumano et al.^12^ has demonstrated the predictive value of soluble TM, t-PAIC, FDP, TAT, PIC, FIB, and D-D as thrombotic markers for septic renal injury. Additionally, Li et al.^13^ has further revealed that TAT, TM, PIC, and t-PAIC can directly reflect the condition of sepsis. However, there is very limited research on thrombotic markers in pregnant women. Some studies have found that monitoring thrombotic markers (such as FDP and D-D) in high-risk pregnant women with gestational diabetes or other conditions can early predict postpartum DVT and enable early prophylaxis.^14^ All these findings indicate the importance of thrombotic markers in the progression of various diseases and their potential predictive value for postpartum complications in women. Thrombotic markers may be closely associated with adverse symptoms or complications.

The six thrombotic markers are used to assess the process of blood clot formation and dissolution.^15^-^17^ In healthy pregnant women, the coagulation and fibrinolytic systems are supposed to maintain a dynamic equilibrium, were blood coagulation and fibrinolysis function normally. In fact, the coagulation and fibrinolytic systems are influenced by various physiological and pathological factors during pregnancy. Specifically, the coagulation system is excessively activated to protect pregnant women from postpartum hemorrhage, which can lead to enhanced coagulation function and HCS. Therefore, several studies have addressed the importance of evaluating hemagglutination parameters during antenatal examinations.^18^-^20^

Weak correlations were observed between TAT levels and maternal age. Likewise, BMI was found to be weakly correlated with FDP and D-D. All this suggests no significant relationship between the general information of pregnant women and their thrombotic status. Therefore, relying solely on routine antenatal examination parameters such as age and BMI may not accurately determine the risk of HCS. However, further establishment of a binary logistic regression model to analyze the influencing factors of HCS revealed that abnormal D-D levels represent a risk factor for HCS in high-risk pregnant women. Moreover, the predictive model established by combining various thrombotic markers had an AUC of 0.757, indicating that the proposed predictive model based on thrombotic markers has a certain predictive value for HCS in pregnant women.

### Limitations:

It includes small sample size, and a single research indicator was observed. In view of this, more patients should be included and thrombotic markers can be combined with other clinical parameters (such as prothrombin time and activated partial thromboplastin time) to enhance the diagnostic accuracy of hypercoagulability in pregnant women by comprehensive evaluation.

## CONCLUSION

The six thrombotic markers TAT, PIC, TM, t-PAIC, D-D, and FDP are useful for the assessment of hypercoagulability in pregnant women, demonstrating predictive value for pregnant women at risk of HCS.

### Authors’ Contributions:

**TY** and **WZ:** Carried out the studies, participated in collecting data, and drafted the manuscript, and are responsible and accountable for the accuracy or integrity of the work.

**YW** and **RC:** Literature search**,** performed the statistical analysis and participated in its design.

**SL:** Participated in acquisition, analysis, or interpretation of data and draft the manuscript, critical review.

All authors read and approved the final manuscript.
